# Multimodal approach to intraventricular hemorrhage using echocardiography, near-infrared spectroscopy, and electrical cardiometry in preterm infants

**DOI:** 10.1038/s41372-025-02544-2

**Published:** 2026-01-05

**Authors:** Aleksandra M. Hibner, Khang Tong, Lin Liu, Ana Morales, Shashank Sanjay, Henry C. Lee, Anup Katheria

**Affiliations:** 1 Division of Neonatology, Department of Pediatrics, University of California, San Diego, California USA; 2 Altman Clinical and Translational Research Institute, University of California, San Diego, California USA; 3 Herbert Wertheim School of Public Health and Human Longevity Science, University of California, San Diego, California USA; 4 Neonatal Research Institute, Sharp Mary Birch Hospital for Women & Newborns, San Diego, California USA

**Keywords:** Outcomes research, Risk factors

## Abstract

**Objective:**

To investigate single versus combination hemodynamic parameters on intraventricular hemorrhage (IVH) or mortality in preterm infants.

**Study design:**

Data from 482 infants under 32 weeks gestational age were analyzed, including cerebral oximetry, mean arterial pressure (MAP), cardiac output, and systemic blood flow within the first 24 h. Wilcoxon Rank-Sum and chi-squared tests compared variables. Multivariable logistic regression and receiver operator curve (ROC) analyses assessed predictive value.

**Results:**

Each additional gestational week was associated with lower odds of IVH (OR = 0.66; 95% CI: 0.57–0.75) and mortality (OR = 0.56; 95% CI: 0.45–0.69). Adjusted for covariates, right ventricular output (RVO) was associated with reduced IVH odds (AOR = 0.996; 95% CI: 0.991–0.999), and higher MAP with reduced mortality (AOR = 0.81; 95% CI: 0.68–0.94). Average NIRS < 74% in 24 h increased mortality risk (OR = 4.16; 95% CI: 1.46–11.0; *P* = 0.005).

**Conclusion:**

Select hemodynamic measures are associated with IVH and death. Combining factors did not enhance early risk prediction.

## Introduction

Preterm infants, particularly those born at or before 32 weeks of gestational age, are at a significantly heightened risk of severe morbidities, including intraventricular hemorrhage (IVH) and death [1]. IVH is believed to result from multiple factors acting on the immature brain, with unstable cerebral blood flow being a significant contributor [2]. Impaired cerebral circulation is an early and prominent characteristic of the onset of IVH. These adverse outcomes are often linked to unstable hemodynamics during the critical early hours of life. The application of non-invasive modalities for improved prediction of IVH has been the subject of research for an extended period.

The use of echocardiography to assess cardiac function and systemic blood flow in preterm infants has been well described [3–5]. Studies have shown that echocardiographic parameters can be predictive of adverse outcomes, such as pulmonary hemorrhage and IVH. Kluckow et al. have established that critically low superior vena cava (SVC) flow on echocardiograms is strongly associated with the development of IVH [6–8]. Additionally, other prospective observational studies have demonstrated that patients who developed IVH generally exhibited lower baseline left ventricular output (LVO) and right ventricular output (RVO), with a trend towards improvement prior to the onset of IVH [9].

Near-infrared spectroscopy (NIRS) measuring cerebral tissue oxygen saturation (StO2) within the first 10 min of life was able to predict which infants would develop severe IVH and death [10]. Monitoring cardiac function [11, 12] and cerebral regional oxygen saturation (rSO2) [13, 14] can identify infants at higher risk for developing IVH before the onset of bleeding. Other noninvasive modalities studied extensively include electrical biosensing technology measuring cardiac output (CO) [15, 16]. Although not a reliable standalone measure of CO in premature infants, it can be valuable for tracking changes in hemodynamic parameters over time, possibly when combined with other modalities [17].

Computerized analysis of continuous blood pressure data in preterm infants has demonstrated that early postnatal hypotension—particularly prolonged periods of low mean arterial blood pressure (MAP)—can predict adverse neurological outcomes such as severe IVH, ischemic cerebral lesions, and increased mortality [18, 19]. However, the predictive value of blood pressure alone is limited by this group’s rapid hemodynamic fluctuations and immature cerebral autoregulation [20, 21], emphasizing the importance of combining continuous blood pressure (BP) monitoring with additional methods like NIRS and functional echocardiography studies to better identify infants at risk for brain injury [8, 22].

Clinical trials have already explored the use of multimodal hemodynamic monitoring in preterm infants, showing that combining various monitoring techniques may provide a more comprehensive assessment of an infant’s hemodynamic status [23]. Limited evidence from studies, employing noninvasive monitoring techniques to predict and manage these hemodynamic abnormalities, has been inconclusive and has not demonstrated a clear benefit [23, 24].

In this study, we aim to investigate whether a multimodal approach, combining various commonly used hemodynamic monitoring modalities, can improve the prediction of IVH and mortality in preterm infants. By integrating data from cerebral oximetry (StO2) measured by near-infrared spectroscopy (NIRS), MAP, CO measured by electrical cardiometry (EC), and systemic blood flow assessed via echocardiography (ECHO), we hypothesized a greater association with death or IVH with the combination of some or all of these modalities.

## Materials/subjects and methods

This study was a retrospective cohort analysis of prospectively collected data from 5 randomized controlled trials conducted at Sharp Mary Birch Hospital for Women and Newborns in San Diego, CA, from January 2013 to December 2024. The original trials received institutional review board (IRB) approval from Sharp HealthCare, and written informed consent was obtained from parents or guardians for all participants. IRB approval was obtained from Sharp HealthCare for this secondary data analysis. Detailed methodological protocols for participant enrollment, inclusion/exclusion criteria, and primary interventions have been previously published [10, 25–28].

### Study population

Eligible participants were preterm infants <32 weeks gestational age admitted to the neonatal unit who had comprehensive cardiovascular and hemodynamic monitoring data, including NIRS, MAP, CO using electrical cardiometry, and ECHO. Hospital records and head ultrasound images available on Synapse were examined to determine IVH severity using Papile’s grading method [29].

### Hemodynamic monitoring

#### Near-infrared spectroscopy

Cerebral oxygen saturation was measured using the FORE-SIGHT Absolute Tissue Monitor (Casmed, Branford, CT) [30] placed on the anterior forehead once the infant was stable (1–3 h of life). Based on calibration against the INVOS device using a neonatal probe [31], a threshold value of 67% was applied for analysis. NIRS data were recorded every 2 s for the first 24 h.

#### Blood pressure monitoring

MAP was continuously measured through an indwelling umbilical arterial catheter and recorded every 2 s. When unavailable, oscillometric cuff measurements were obtained every 2–6 h.

#### Electrical cardiometry

Electrical cardiometry sensors (ICON device, Cardiotronic, La Jolla, CA) were placed on the infant’s head, neck, axilla, and inguinal canal to measure stroke volume and cardiac output [32, 33]. Data were recorded every 2 s for 24 h using a digital acquisition system (MP150, Biopac, Goleta CA). Compromised cardiac output was defined as <150 ml/kg/min [33].

#### Echocardiography

A single functional echocardiogram was performed within the first 12 h of life using Vivid E9 (GE Healthcare, Wauwatosa, WI) to assess SVC flow, LVO, and RVO. A pediatric cardiologist reviewed all scans for structural abnormalities.

#### Head ultrasound

**Fig. 1:**
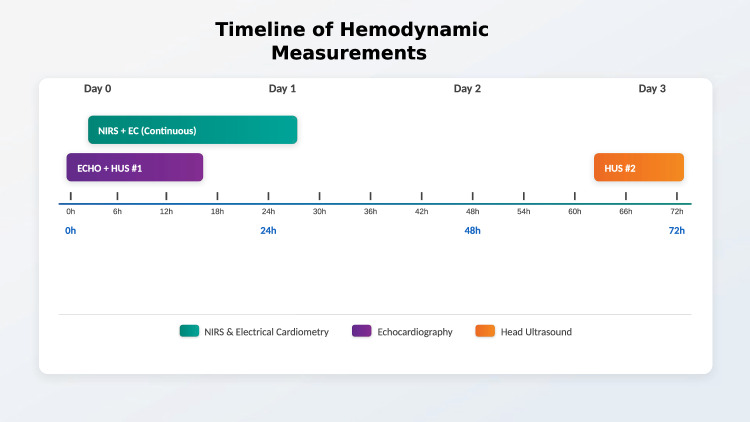
Timeline of hemodynamic measurements in relation to intraventricular hemorrhage detection. The figure shows the temporal relationship of continuous NIRS and electrical cardiometry monitoring, echocardiography assessments, and head ultrasound examinations over the first 72 h of life.

All infants underwent transcranial ultrasound within 12 h of life, 72 h, and 7 days. Radiologists blinded to hemodynamic parameters interpreted results using Papile’s grading [29]. The timeline of all hemodynamic measurements relative to IVH detection on head ultrasound is shown below (Fig. 1).

### Data collection

Demographic and baseline data were collected, including gestational age, birth weight, antenatal steroid use, delivery method, sex, chorioamnionitis, and pregnancy-induced hypertension. The primary outcomes for this analysis were IVH severity and mortality. Since this was a convenience sample, an a priori sample size was not calculated. Relevant maternal and neonatal medical information was collected from the electronic medical records and recorded using the REDCap electronic database application hosted at SHARP Healthcare (REDCap v5.7.1 2014, Vanderbilt University). Additional variables comprised mean arterial pressure, cerebral NIRS readings, and CO. All data were collected in the first 24 h of life. The duration of cerebral hypoxia during which NIRS dropped below 67% and low cardiac output, when electrocardiometry measurements fell under 150 ml/kg/min were extracted using Excel macros and Visual Basic for Applications (VBA). Echocardiography was conducted once within the first 24 h of life to assess SVC, as well as both RVO and LVO.

### Statistical analysis

Continuous variables were summarized as mean and standard deviation (SD), while categorical variables were presented as counts and percentages. Variable summaries were stratified by three binary outcomes: any IVH, severe IVH, and death, and compared using Wilcoxon Rank-Sum and chi-squared tests for continuous and categorical variables, respectively. Univariable and multivariable logistic regressions were conducted to examine the association between potential predictors and outcomes. Multivariable analyses were conducted for two separate predictor sets: average/raw measurements set including mean over 24 h (or raw value for echocardiograms) for MAP, NIRS, CO by electrical cardiometry, SVC flow, RVO, and LVO; abnormal time set including total time over 24 h with abnormal values (NIRS < 67, cardiac output < 150 ml/kg/min). Additionally, prenatal steroids and chorioamnionitis were considered as controlling variables for both analysis sets, while all analyses were controlled for gestational age (GA). Birth weight was summarized but excluded from regression models due to collinearity with gestational age. The initial multivariable logistic regression model included potential predictors in each set with a univariable *P* < 0.20. Backward selection was used to iteratively remove the variable with the highest *P*-value and refit until all remaining variables had *P* < 0.10. This process was performed separately for any IVH, severe IVH, and death outcomes.

In exploratory analyses, ROC curves were generated for each predictor to evaluate discriminatory ability for any-IVH and mortality. The optimal cutpoint for each predictor was determined using Youden’s index, area under the curve (AUC), the direction of association, sensitivity, specificity, positive predictive value, and negative predictive value calculated. Predictors were then dichotomized at the optimal cutpoint, and their associations with each outcome were evaluated using univariable and multivariable logistic regression methods as described above to analyze these predictors on the original scale. All statistical analyses were performed using R Studio, version 4.3.0 (R Foundation for Statistical Computing), and a two-tailed *P* < 0.05 was considered statistically significant.

While multiple measures of CO were included in initial models (SVC flow, LVO, RVO, and EC-derived CO), backward selection naturally addressed potential multicollinearity by retaining only the most independently predictive variables for each outcome.

## Results

Among the 482 infants born at <32 weeks’ GA, gestational age ranged from 23.0 to 31.9 weeks (mean ± SD: 28.0 ± 2.0 weeks), and birth weight ranged from 420 to 2180 g (mean ± SD: 1118 ± 383 g). Fifty-four infants (11.2%) developed any-grade IVH, and 15 (3.1%) had severe IVH (grade 3 or 4). (Table 1). Baseline characteristics indicated that infants with IVH were significantly more preterm, had lower birth weights, and a higher incidence of chorioamnionitis compared to those without IVH (all *P* ≤ 0.001, Table 1). Severe IVH and mortality were associated with lower GA, MAP, and birth weights (all *P* < 0.01, Tables 2 and 3). GA was found to be strongly associated with each outcome, with each additional week of GA being associated with a 34% (OR = 0.66; 95% CI: 0.57–0.75), 42% (OR = 0.58; 95% CI: 0.44–0.76), and 44% (OR = 0.56; 95% CI: 0.45–0.69) reduction in the odds of any IVH, severe IVH, and mortality respectively (all *P* < 0.001). Unadjusted and adjusted analyses yielded consistent results for all three outcomes: any-grade IVH, severe IVH, and mortality.

**Table 1:** Maternal and neonatal characteristics by IVH status.

Variable	Overall (*n* = 482)	No IVH (*n* = 428)	Any IVH (*n* = 54)	*P*-value
Gestational age (weeks), Mean ± SD	28.0 ± 2.0	28.4 ± 2.3	26.2 ± 2.2	<0.001*
Birth weight (grams), Mean ± SD	1118 ± 383	1148 ± 381	888 ± 324	<0.001*
Antenatal steroid (completed course), *n (%)*	396 (82%)	355 (83%)	41 (76%)	0.28
Cesarean section, *n (%)*	413 (85%)	372 (87%)	41 (76%)	0.03*
Female, *n (%)*	247 (51%)	219 (51%)	28 (55%)	0.92
Chorioamnionitis, *n (%)*	116 (24%)	93 (22%)	23 (43%)	0.001*
Pregnancy-induced hypertension, *n (%)*	122 (25%)	116 (27%)	6 (11%)	0.01*
IVH Grade 1, *n (%)*	25 (5.1%)	–	25 (46%)	
				–
Moderate IVH Grade 2, *n (%)*	14 (2.9%)	–	14 (26%)	
				–
Severe IVH Grade 3 & 4, *n (%)*	15 (3.1%)	–	15 (28%)	
				–
Death, *n (%)*	27 (6%)	15 (3.5%)	12 (22%)	<0.0001*

*SD* Standard Deviation, *IVH* Intraventricular Hemorrhage.

*Indicates statistically significant at *p* < 0.05.

**Table 2:** Predictor variables stratified by severe IVH (Mean ± SD).

Variable	No IVH/	Severe IVH	OVERALL	*P* value
	Grade 1, 2 IVH (*n* = 467)	(*n* = 15)	(*n *= 482)	
Gestational age (weeks)	28.3 ± 2.4	25.4 ± 1.8	28.2 ± 2.4	<0.001
Birth weight (g)	1131 ± 381	751 ± 250	1119 ± 384	<0.001
Mean arterial blood pressure (mmHg)	35.1 ± 9.7	29.4 ± 6.98	35.0 ± 9.7	0.006
Near infrared spectroscopy average (NIRS) (%)	80.4 ± 6.5	77.6 ± 13.7	80.3 ± 6.8	0.04
Cardiac output average (ml/kg/min)	242.5 ± 472	267.9 ± 75.6	243.2 ± 466	0.03
Superior vena cava flow (SVC)	86.9 ± 29.6	61.5 ± 36.8	86.2 ± 29.9	0.1
(ml/kg/min)				
Right ventricular function (RVO) (ml/kg/min)	252.9 ± 89.9	195.3 ± 82.6	254.9 ± 90.2	0.026
Left ventricular function (LVO) (ml/kg/min)	201.7 ± 67.9	160.6 ± 56.9	200.3 ± 67.85	0.046

*P*-values for continuous variables come from the Wilcoxon rank-sum test, and for categorical variables from the chi-squared test.

**Table 3:** Predictor variables stratified by death (Mean ± SD).

Variable	No death	Death	Overall	*P* value
	(*n* = 455)	(*n* = 27)	(*n* = 482)	
Gestational age (weeks)	28.3 ± 2.3	25.4 ± 1.7	28.2 ± 2.4	<0.001
Birth weight (g)	1147 ± 374	656 ± 232	1119 ± 384	<0.001
Mean arterial blood pressure (mmHg)	35.3 ± 9.7	30.1 ± 7.7	35.0 ± 9.7	0.002
Near infrared spectroscopy average (NIRS) (%)	80.3 ± 6.6	80.0 ± 10.0	80.3 ± 6.8	0.35
Cardiac output average (ml/kg/min)	243.0 ± 475.0	247.2 ± 100.0	243.2 ± 466.0	0.28
Superior vena cava flow (SVC)	86.7 ± 29.4	73.8 ± 28.2	86.2 ± 29.9	0.3
(ml/kg/min)				
Right ventricular function (RVO) (ml/kg/min)	253.4 ± 85.6	276.9 ± 143.0	254.9 ± 90.2	0.8
Left ventricular function (LVO) (ml/kg/min)	199.0 ± 67.8	219.5 ± 56.9	219.5 ± 56.9	0.046

*P*-values for continuous variables come from the Wilcoxon rank-sum test, and for categorical variables from the chi-squared test.

Any-grade IVH: A 10 mL/kg/min increase in RVO was associated with a 4% reduction in the odds of any-grade IVH (AOR = 0.96 (0.92–0.99), *P* = 0.03). No variables from the time with abnormal measurements set were retained in the final models for any-grade IVH after adjustment for GA.

Severe IVH: After adjusting for GA, a 1 mL/kg/min increase in SVC flow was associated with a 4% reduction in the odds of severe IVH (AOR = 0.96 (95% CI: 0.93–0.99), *P* = 0.02) (Table 4). Each additional minute with abnormal NIRS (<67%) was associated with a 0.2% increase in the odds of severe IVH (AOR = 1.002 (1.001–1.003), *P* = 0.004).

**Table 4:** Adjusted odds ratio on the effect of significant hemodynamic parameters for each primary outcome from a multivariable logistic regression model.

	Adjusted odds ratio (95% CI)	*P*-value
Any-Grade IVH^a^		
*Average/raw measurements set*		
RVO^1^	0.96 (0.92, 0.99)	0.03
Severe IVH		
*Average/raw measurements set*		
SVC^1^	0.96 (0.93, 0.99)	0.02
*Abnormal time set*		
Number of minutes NIRS < 67%^1^	1.002 (1.001, 1.003)	0.004
Mortality^a^		
*Average/raw measurements set*		
MAP^2^	0.81 (0.68, 0.94)	0.01
SVC^3^	0.96 (0.92, 0.99)	0.02

Adjusted by: ^1^GA, ^2^GA, SVC, prenatal steroid exposure, ^3^GA, MAP, prenatal steroid exposure.

*CI* Confidence interval.

^a^No significant hemodynamic parameters from the abnormal time set.

Mortality: Each 1 mmHg increase in MAP over the first 24 h was associated with a 19% reduction in the odds of death (OR = 0.81; 95% CI: 0.68–0.94; *P* = 0.013) after adjusting for GA, SVC flow, and prenatal steroid exposure. No variables from the time with abnormal measurements set were significant predictors of mortality.

Hemodynamic parameters were analyzed collectively using a multivariable regression model. Gestational age emerged as a strong predictor of both outcomes, and after adjusting for it, other parameters did not contribute significantly to outcome prediction. In exploratory analyses, ROC curve analysis demonstrated that GA alone had the highest sensitivity and specificity for predicting both IVH and mortality (Supplementary Tables 1, 2). In a separate multivariable model using ROC-derived cut points, after controlling for GA, having an average NIRS value < 74% during the first 24 h significantly increased the odds of mortality (OR = 4.16; 95% CI: 1.46–11.0; *P* = 0.005).

While echocardiography-derived measures (SVC flow, RVO, LVO) showed lower values in severe IVH as expected, electrocardiometry paradoxically demonstrated higher CO in the severe IVH group (Table 2). This discrepancy and the large standard deviation in the no-IVH group emphasize electrical cardiometry’s measurement limitations in this population.

## Discussion

In this extensive retrospective cohort of preterm infants born before 32 weeks’ GA, we investigated whether incorporating multimodal hemodynamic monitoring—including ECHO, NIRS, MAP, and EC—could facilitate the earlier identification of infants at heightened risk for IVH and mortality. While parameters such as NIRS and SVC flow exhibited initial associations with lower IVH and mortality rates, these correlations diminished after adjusting for GA, indicating the predominant influence of GA in predicting outcomes.

Neonatologists increasingly recognize that assessing perfusion and tissue oxygen delivery requires more than blood pressure and oxygen saturation [13]. Prior research supports a multifactorial, physiology-based approach using contemporary monitoring technologies and individual patient profiles. The association between higher MAP and improved survival is biologically plausible given the dependence of cerebral perfusion on systemic BP in extremely preterm infants with immature autoregulation. Furthermore, treatments targeting hypotension did not enhance survival or neurodevelopmental outcomes and were associated with an increased risk of adverse events [34]. Our findings align with prior studies showing lower MAP in infants with IVH or who died, though we did not evaluate interventions or therapeutic choices based on BP values [35]. Recent literature cautions against relying solely on MAP, advocating for broader assessment including systolic and diastolic pressures and BP variability, which may better reflect cerebral hemodynamics. Jiang et al. identified a correlation between high BP variability and the resistance index of the anterior cerebral artery in a cohort of 92 infants, suggesting a link between IVH and these factors [36].

Echocardiography remains valuable for detecting low-flow states. Prior studies have linked low SVC flow to higher IVH incidence, though our cohort did not show significant changes in LVO preceding IVH [7, 9, 12]. Emerging evidence suggests that early, targeted neonatal echocardiographic (TNE) assessment—when integrated into clinical decision-making—can improve outcomes in preterm infants by identifying hemodynamic instability and guiding timely interventions [11]. Studies have linked early TNE and hemodynamic screening with reduced mortality and lower rates of severe IVH, supporting the value of serial, physiology-guided echocardiography in optimizing care for this high-risk population [37]. NIRS values were not significantly different across groups, but secondary analysis suggested that sustained values below 74% may be associated with mortality. However, large trials like SafeBoosC-III have not demonstrated improved outcomes using NIRS-guided management [38]. A study by Alderliesten et al. found that MAP less than GA (in weeks) was not associated with lower StO2 or neurodevelopmental outcome scores. However, low StO_2_ was associated with lower neurodevelopmental outcome scores regardless of MAP [39]. While electrical cardiometry proved feasible for continuous monitoring, its weak association with IVH and mortality—consistent with prior studies—suggests limited prognostic utility [17, 40–42].

Despite limited predictive value in our study, multimodal monitoring offers potential merits by providing comprehensive physiological assessment across cardiovascular dimensions and identifying distinct hemodynamic phenotypes requiring different management strategies. For example, adequate BP with low CO and decreased StO2 represents a different state than low BP with preserved perfusion—distinctions impossible with single-modality monitoring. Beyond outcome prediction, multimodal assessment may guide treatment decisions, monitor therapeutic responses, and identify risks for other complications. Its value may lie in dynamic, real-time assessment rather than static prediction—an aspect prospective implementation studies should address.

### Strengths and limitations

A key strength of our study is its substantial sample size and the use of prospectively collected physiological data from multiple randomized trials. This comprehensive hemodynamic approach, coupled with multimodal assessment, enhances both data quality and generalizability. Furthermore, the study employed time-resolved continuous data, such as second-by-second NIRS and electrocardiography readings, enabling detailed analysis of not only average values but also the duration of periods below clinically relevant thresholds.

However, several limitations should be acknowledged. First, despite using prospectively collected data, the retrospective nature of the analysis may introduce bias from unmeasured confounders. Second, echocardiographic measurements were limited to a single time point within the first 12 h, potentially missing critical fluctuations that could be relevant to the development of IVH. Third, despite calibration efforts, variability between devices and operators in NIRS and electrocardiography readings may impact data accuracy.

A significant limitation is the absence of systematically collected data on clinical interventions and their temporal relationship to hemodynamic measurements. Preterm infants in this gestational age range routinely undergo multiple therapeutic interventions during the first 24 h of life—including delivery room resuscitation, intubation, surfactant administration, umbilical catheter placement, inotropic support, and fluid boluses—all of which can substantially influence hemodynamic parameters. As this was a secondary analysis of data from randomized controlled trials with different primary objectives, we did not capture detailed information on the timing, type, or dosing of these interventions.

Additional limitations include excluding MAP from our ‘abnormal time set’ analysis. MAP was analyzed as a continuous variable rather than with time-based thresholds due to the absence of consensus on hypotension definitions in extremely preterm infants, with varied cutoffs proposed in the literature.

#### Clinical implications and future research

This study’s findings have substantial implications for contemporary clinical practice and the future development of hemodynamic monitoring in the neonatal intensive care unit (NICU). Our findings corroborate GA as a pivotal factor in determining the risk of preterm infants. While individual hemodynamic parameters, such as RVO and SVC flow, provide valuable insights into cardiovascular status, as predictors of IVH or mortality when adjusting for GA. Ongoing prospective clinical trials such as the PIONIRS trial (NCT05708105) could advance research in premature infants through combined NIRS and echocardiography assessments. This could assist in guiding timely neuroprotective treatments and shaping future prevention strategies. Real-time, individualized hemodynamic stability assessment remains indispensable for guiding interventions, identifying evolving instability, and potentially mitigating secondary brain injury.

Future investigations should address several key areas to advance hemodynamic monitoring in preterm infants. Establishing evidence-based blood pressure thresholds would enable time-based MAP analyses comparable to those performed for NIRS and cardiac output. Additionally, incorporating detailed cardiovascular intervention data, including inotrope administration, timing, and dosing, would allow for more comprehensive assessment of how therapeutic interventions influence hemodynamic parameters and outcomes. Such studies would better elucidate the independent predictive value of multimodal hemodynamic monitoring and guide clinical decision-making.

There should be a focus on dynamic, time-dependent modeling of hemodynamic changes rather than single-value cutoffs. Combining machine learning and real-time analytics with NICU bedside monitoring systems may facilitate more accurate risk stratification and better intervention timing. Moreover, randomized trials are imperative to verify whether clinical management based on multimodal hemodynamic feedback leads to improved outcomes.

## Conclusion

Routinely combining multiple hemodynamic parameters for early outcome prediction may not be justified in all preterm infants. Individualized, real-time monitoring and the integration of advanced analytics represent a promising strategy for improving outcomes in the most vulnerable neonatal populations, but need further testing in larger randomized controlled trials.

## Supplementary information

Supplementary Table 1.

Supplementary Table 2.

## Data Availability

The de-identified clinical datasets supporting the conclusions of this study are available from the corresponding author upon reasonable request, subject to institutional review board approval and data use agreements to protect patient confidentiality.
